# Toward Integrating Intranasal Esketamine with Traumatic-Memory Psychotherapy in Treatment-Resistant Depression: A Narrative Review and Feasibility-Oriented Protocol Proposal

**DOI:** 10.3390/bs16050771

**Published:** 2026-05-14

**Authors:** Fabiola Raffone, Carlo Ignazio Cattaneo, Enrico Pessina, Azzurra Martini, Vassilis Martiadis

**Affiliations:** 1 Department of Psychiatry, University of Campania “Luigi Vanvitelli”, 81100 Naples, Italy; 2 Department of Mental Health, Asl Napoli 1 Centro, 80125 Naples, Italy; 3 Department of Mental Health, Asl Biella, 13900 Biella, Italy; 4 Department of Mental Health, Asl Cuneo 2, 12042 Cuneo, Italy

**Keywords:** esketamine, ketamine, treatment-resistant depression, post-traumatic stress disorder, traumatic memories, intrusive memories, psychotherapy, imagery rescripting, memory reconsolidation, intranasal administration

## Abstract

Trauma-related autobiographical memories can manifest as involuntary, vivid, emotionally charged intrusions that perpetuate avoidance, negative emotions, and functional impairment. While these memories are central to post-traumatic stress disorder (PTSD), they also occur across diagnoses and are often reported in depressive disorders, including treatment-resistant depression (TRD). Although trauma-focused psychotherapies are effective, their routine implementation can be limited by dropout, residual symptoms, and difficulty engaging patients with severe depression, dissociation, or complex comorbidities. Intranasal esketamine is an approved rapid-acting treatment for TRD and has been hypothesized to create transient conditions that may facilitate psychotherapeutic work on traumatic memories. This narrative review synthesizes clinical and translational evidence on ketamine and esketamine for PTSD and trauma-related symptoms, with particular attention to the distinction between intravenous ketamine studies, intranasal esketamine data, and studies combining these compounds with psychotherapy. Currently, the most robust evidence in this area comes from three randomised trials of intravenous ketamine for PTSD. In contrast, data on intranasal esketamine and psychotherapy-combination approaches are mainly from pilot studies, retrospective analyses, or case reports. We additionally propose a pragmatic, feasibility-oriented protocol integrating intranasal esketamine with a structured traumatic-memory intervention for TRD patients with clinically relevant trauma-memory symptoms. The novelty of the proposal does not lie in claiming efficacy, but in specifying a standardised imagery rescripting module and predefining two timing hypotheses. The proposal targets patients with TRD with relevant trauma-memory symptoms, and it embeds the intervention within existing esketamine-care infrastructure. Overall, the available literature supports mechanistic plausibility and preliminary feasibility more than clinical efficacy. The evidence base remains small, heterogeneous, and largely uncontrolled, and controlled studies are needed before efficacy claims can be made.

## 1. Introduction

Traumatic experiences are encoded as autobiographical memories that can later be reactivated as distressing intrusions, flashbacks, nightmares, or intrusive mental imagery. These intrusive images and memories have been conceptualized as transdiagnostic phenomena that contribute to symptom persistence across psychiatric disorders (Brewin et al., 2010; Iyadurai et al., 2019). Meta-analytic evidence confirms that intrusive memories are prevalent in adult depression as well, indicating that memory intrusions are not exclusive to PTSD and may be a clinically relevant target in depressive disorders (Payne et al., 2019).

In PTSD, traumatic memories are characterized by heightened sensory-emotional salience and are maintained by maladaptive appraisals and avoidance-based coping strategies. The cognitive model of PTSD (Ehlers & Clark, 2000) emphasizes the interplay between poorly elaborated trauma memory representations and catastrophic interpretations that sustain a persistent sense of current threat. Although trauma-focused psychotherapies, including cognitive processing therapy, prolonged exposure, and EMDR, demonstrate robust efficacy, their effectiveness in routine care can be limited by low uptake, clinically meaningful dropout rates of approximately 20–30%, and residual symptom burden after treatment completion, especially in complex cases (Lewis et al., 2020).

Trauma exposure is also highly relevant to TRD. A recent systematic review documented that early or recent trauma exposure is associated with treatment resistance in depression, supporting the need to address trauma-related mechanisms even when full PTSD diagnostic criteria are not met (Fantasia et al., 2025). In this context, traumatic-memory symptoms (e.g., intrusions, avoidance, and affective reactivity to reminders) may represent a mechanistically informed target for combined pharmacological and psychotherapeutic strategies.

Intranasal esketamine, a rapid-acting glutamatergic antidepressant, has been approved for TRD under supervised administration and monitoring (Food and Drug Administration, 2025). Randomised clinical trials have demonstrated rapid and significant improvements in depressive symptoms when esketamine is used adjunctively with an oral antidepressant (Daly et al., 2018; Popova et al., 2019), including in older adults (Ochs-Ross et al., 2020), and more recently in a monotherapy regimen (Janik et al., 2025). Real-world studies further support feasibility and a manageable safety profile in naturalistic settings (Martinotti et al., 2022). Multidisciplinary care frameworks that integrate psychiatric, psychotherapeutic, and nursing competencies have been developed for esketamine programs in routine settings (Martiadis et al., 2026). These frameworks provide a relevant implementation model for the combined protocol proposed in this paper.

Beyond their established antidepressant effects, ketamine and esketamine may modulate learning and memory processes relevant to trauma-related symptoms. Controlled trials of intravenous ketamine for chronic PTSD have reported rapid reductions in symptom severity (Feder et al., 2014, 2021; Abdallah et al., 2022). By contrast, data on intranasal esketamine in TRD patients with comorbid PTSD or trauma-memory symptoms remain limited to small pilot or observational reports, including a retrospective case series documenting transient trauma re-experiencing episodes that require careful clinical management (Rothärmel et al., 2022, 2026). Qualitative research on patient narratives of esketamine-induced dissociation has illuminated the subjective phenomenological landscape of these states, with potential implications for therapeutic framing and preparation (Olivola et al., 2026).

Taken together, these observations suggest that trauma-memory symptoms may be a clinically relevant but understudied target in TRD. However, direct evidence that intranasal esketamine enhances trauma-focused psychotherapy is currently lacking, and mechanistic inferences from intravenous ketamine or preclinical work should not be treated as evidence of clinical efficacy in this specific context.

Against this background, the aims of this narrative review are twofold: (i) to summarize evidence linking ketamine/esketamine to PTSD symptoms and traumatic-memory processes, with explicit attention to differences between intravenous ketamine, intranasal esketamine, and psychotherapy-combination studies; and (ii) to propose a pragmatic, feasibility-oriented protocol integrating intranasal esketamine with structured traumatic-memory psychotherapy for TRD patients presenting with clinically relevant trauma-memory symptoms. The overarching goal is not to infer efficacy from the current literature, but to outline a cautious, hypothesis-generating research agenda for future controlled studies.

## 2. Materials and Methods

### 2.1. Databases and Search Strategy

We conducted a narrative review to synthesize evidence on the use of intranasal esketamine and intravenous ketamine as a mechanistic and clinical comparator in relation to traumatic memories, PTSD symptoms, and psychotherapy approaches targeting trauma-related memory processes. In the interest of transparency, we are reporting the databases, search strings, dates and broad selection steps. However, this was a structured narrative search undertaken to inform a protocol framework focused on feasibility, not a formal systematic or scoping review. Consequently, the review was not protocol-registered, did not adhere to PRISMA reporting standards and was not designed for quantitative synthesis.

The review aimed to inform a protocol proposal, emphasizing clinical feasibility and translational relevance over exhaustive quantitative pooling. It was intended to identify patterns, gaps, and design considerations rather than to estimate pooled effects or support causal inferences. We searched PubMed/MEDLINE and Scopus from database inception to 25 January 2026. ClinicalTrials.gov was additionally consulted to identify registered trials combining ketamine or esketamine with psychotherapy for PTSD or trauma-related symptoms; no unpublished trial data meeting inclusion criteria were identified, and this source did not contribute records to the narrative synthesis. Reference lists of key articles were manually searched for additional foundational and mechanistic references.

The PubMed/MEDLINE search string was:

“(esketamine OR ketamine) AND (posttraumatic stress disorder OR PTSD OR trauma OR traumatic) AND (memory OR memories OR intrusive OR flashback OR reconsolidation OR extinction OR psychotherapy OR EMDR OR exposure OR written exposure OR imagery rescripting)”

The equivalent Scopus search string was:

“TITLE-ABS-KEY((esketamine OR ketamine) AND (“posttraumatic stress disorder” OR PTSD OR trauma OR traumatic) AND (memory OR memories OR intrusive OR flashback OR reconsolidation OR extinction OR psychotherapy OR EMDR OR “exposure therapy” OR “written exposure” OR “imagery rescripting”))”.

### 2.2. Eligibility Criteria

We included the following: (i) randomized controlled trials, open-label studies, observational studies, case series, and case reports involving ketamine or esketamine for PTSD or trauma-related symptoms, (ii) studies explicitly addressing traumatic memories, flashbacks, intrusive memories, or trauma-related learning processes, and (iii) studies combining ketamine or esketamine with a structured psychotherapy or a psychotherapeutic framework targeting trauma. We excluded animal-only studies and papers without accessible full texts when key methodological details could not be verified.

### 2.3. Study Selection and Data Extraction

Two reviewers independently and non-blindedly screened titles and abstracts and subsequently assessed potentially eligible articles in full text. Disagreements were resolved by consensus discussion and recorded in the review notes. The following information was extracted: study design; population characteristics; ketamine/esketamine route and dosing; psychotherapy type and timing relative to dosing; primary outcomes; key safety observations; and memory-specific findings. The combined database search yielded 906 records (PubMed/MEDLINE: 371; Scopus: 535). After removal of duplicates (*n* = 220), 686 records were screened by title and abstract. Of these, 633 were excluded as out of scope, animal-only, or lacking accessible full texts. Fifty-three records were assessed in full text for eligibility; 25 were subsequently excluded (insufficient methodological reporting, *n* = 12; absence of memory-related or PTSD-relevant outcomes, *n* = 9; full text not retrievable, *n* = 4). A total of 28 records were included in the narrative synthesis from the database search. Nine additional references were identified through manual searching of reference lists of key articles; these were cited where they directly supported the conceptual or mechanistic framework. Thus, 37 references directly informed the narrative synthesis. This review was not conducted or reported as a PRISMA systematic review. Potential selection bias inherent to the narrative design is acknowledged in Section 8.6.

### 2.4. Synthesis Approach

Due to the heterogeneity of study designs and interventions, the findings were synthesized narratively. Higher-level evidence was prioritized, and case series were used mainly to contextualize feasibility, phenomenology, and safety. The synthesis was organized around four thematic areas: (i) the effects of ketamine on PTSD symptoms, (ii) medication-enhanced psychotherapy studies, (iii) esketamine for TRD with comorbid PTSD or trauma-memory symptoms, and (iv) the implications of a structured protocol targeting traumatic memories. Accordingly, the review should be interpreted as hypothesis-generating rather than confirmatory. Due to the significant variation in study designs and interventions, no formal risk-of-bias instrument was applied at the study level. Instead, the studies were categorised according to their level of evidence (RCT, open-label/observational or retrospective/case-based) and appraised narratively with regard to sample size, control condition, intervention heterogeneity, follow-up duration and key threats to causal inference.

## 3. Intranasal Esketamine in Treatment-Resistant Depression: Clinical Context and Relevance to Trauma-Memory Work

Due to risks of sedation, dissociation, transient blood pressure increases, and potential for misuse, intranasal esketamine is administered under direct medical supervision with mandatory post-dose monitoring (Food and Drug Administration, 2025). Randomized clinical trials have established rapid antidepressant effects when esketamine is used adjunctively with an oral antidepressant (Daly et al., 2018; Popova et al., 2019), including in the TRANSFORM-3 Phase III trial in older adults (Ochs-Ross et al., 2020), and more recently in a monotherapy regimen (Janik et al., 2025). Real-world studies, including the multicentric REAL-ESK study in Italian mental health services, demonstrate significant symptomatic improvement and a manageable safety profile across patients with diverse psychiatric comorbidities (Martinotti et al., 2022). Patient-perspective data from the REAL-ESKperience study indicate high overall satisfaction (Di Nicola et al., 2025), and secondary analyses from the REAL-ESK group demonstrate reductions in anhedonic symptoms (D’Andrea et al., 2025), a domain particularly relevant to psychotherapy engagement.

Integrated, multidisciplinary care frameworks that combine psychiatric, psychotherapeutic, and nursing roles have been developed for intranasal esketamine programs in routine settings (Martiadis et al., 2026). This framework provides a directly applicable implementation model for the combined protocol proposed in this paper because it establishes the organizational and competency prerequisites for incorporating structured psychotherapy within an esketamine clinic.

Qualitative research on patient narratives of esketamine-induced dissociation in TRD has revealed a complex and heterogeneous phenomenological landscape, encompassing themes of detachment, altered bodily experience, and transient emotional accessibility (Olivola et al., 2026). This evidence underscores the importance of individualized preparation and post-session integration, and suggests that the subjective meaning patients attribute to dissociative experiences may be therapeutically relevant when appropriately contextualized. However, altered states may also increase vulnerability to distressing intrusions. Real-world evidence indicates that safety signals related to suicidality may vary across patient subgroups, including by gender (Martiadis et al., 2025), underscoring the importance of systematic monitoring.

## 4. Mechanistic Rationale for Esketamine-Enhanced Traumatic-Memory Psychotherapy

The rationale for combining esketamine with trauma-focused psychotherapy derives primarily from preclinical work and from broader evidence that ketamine-class compounds modulate neurobiological processes involved in learning, synaptic plasticity, and the updating of maladaptive memory representations. At the synaptic level, ketamine and esketamine primarily act as noncompetitive antagonists of N-methyl-D-aspartate (NMDA)-type glutamate receptors. Acute NMDA blockade triggers downstream molecular events, including transient glutamate surges, AMPA receptor activation, and phosphorylation within the BDNF/TrkB signaling pathway. These events converge on mTORC1 activation and rapid synaptic protein synthesis, and have been associated with enhanced dendritic spine density and synaptic strength in prefrontal cortical circuits within hours (Li et al., 2010; Autry et al., 2011; Duman et al., 2016). From a memory science perspective, reconsolidation theory offers a plausible translational framework. Upon retrieval, consolidated memory traces enter a transient labile state, during which they can be updated before restabilisation. NMDA receptor activity appears to be one molecular gate in these processes (Nader et al., 2000; Ben Mamou et al., 2006). However, NMDA antagonism should not be assumed to uniformly facilitate therapeutic learning, including extinction-related learning, as its effects may vary according to timing, dosage, task demands and memory type. Furthermore, complex autobiographical trauma memories are not equivalent to simple conditioned fear paradigms. Therefore, direct evidence linking the molecular effects of intranasal esketamine in humans to a clinically usable psychotherapeutic window is lacking, and the mechanistic account presented here should be regarded as plausible but provisional.

At the clinical level, intravenous ketamine has demonstrated rapid reductions in PTSD symptom severity across randomised trials (Feder et al., 2014, 2021; Abdallah et al., 2022), suggesting that glutamatergic interventions may influence trauma-related symptom circuits. Neuroimaging evidence also supports durable neurobiological effects following ketamine administration (Duek et al., 2023). Case-based studies of specific depressive phenotypes have interpreted esketamine-induced disembodiment as a possible window of psychological accessibility that may be supported within a psychotherapeutic context (Sarasso et al., 2024). These findings are informative, but they do not establish that intranasal esketamine augments psychotherapy in TRD. Consequently, any combined protocol must prioritize structured preparation, grounding procedures, and cautious exploratory framing.

## 5. Evidence Base: Ketamine/Esketamine, PTSD Symptoms, and Psychotherapy Integration

### 5.1. Intravenous Ketamine for PTSD Symptoms

Randomised controlled trials provide the most rigorous clinical evidence that ketamine can rapidly reduce the severity of PTSD symptoms. In a landmark double-blind randomised controlled trial (RCT) (Feder et al., 2014), 41 adults with chronic PTSD were randomized to receive either a single intravenous infusion of ketamine (0.5 mg/kg over 40 min) or an active control (midazolam, 0.045 mg/kg). Ketamine produced a significantly greater reduction in PTSD symptom severity after 24 h, with 46% of participants treated with ketamine meeting response criteria on the CAPS, compared to 20% of those in the control group. Reductions in intrusion and avoidance symptoms were observed alongside reductions in hyperarousal. Adverse effects were transient and included dissociative symptoms and mild hemodynamic changes; no serious adverse events were reported. A subsequent RCT (Feder et al., 2021) enrolled 30 participants with chronic PTSD in a crossover design, comparing repeated ketamine infusions (six infusions over two weeks at 0.5 mg/kg) with an active midazolam control. Ketamine produced sustained reductions in PTSD symptom severity across multiple sessions, with approximately 67% of participants meeting response criteria after the full infusion series, although symptom durability beyond the acute treatment period remains incompletely characterized. A multicentre, double-blind, dose-ranging RCT (Abdallah et al., 2022) in veterans and active-duty military personnel with antidepressant-resistant PTSD symptoms evaluated three ketamine doses (0.2, 0.5, and 1.0 mg/kg) against saline placebo. Dose-related effects were observed in terms of reducing PTSD symptoms, supporting proof-of-concept for a glutamatergic mechanism. However, these data derive from intravenous administration in chronic PTSD samples and should not be extrapolated directly to intranasal esketamine, to TRD populations, or to psychotherapy-augmentation effects.

### 5.2. Ketamine-Assisted Psychotherapy and Memory-Focused Interventions

Several groups have explored whether ketamine can be paired with structured psychotherapies that directly engage trauma memories or extinction/reconsolidation processes. In a proof-of-concept study, repeated subanesthetic intravenous ketamine was combined with prolonged exposure therapy in veterans with PTSD, showing feasibility and clinically meaningful symptom improvement in a small open-label sample (Shiroma et al., 2020). In a subsequent open-label clinical trial, 14 patients with chronic PTSD began treatment and 13 completed a combined protocol of six intravenous ketamine infusions plus five Written Exposure Therapy sessions over approximately three weeks, with large reductions in PTSD severity observed through follow-up (Feder et al., 2025). Ketamine-assisted EMDR has also been described in a retrospective clinical chart review of eight clients with PTSD receiving low-dose sublingual ketamine during EMDR reprocessing, with significant reductions in PTSD symptoms and functional impairment and minimal adverse effects (Topel & Ciccone, 2025). Taken together, these studies suggest feasibility and mechanistic plausibility, but they remain preliminary because sample sizes are small and controlled comparisons are lacking. Accordingly, the existing combination literature should be viewed as feasibility- and hypothesis-generating rather than efficacy-establishing.

### 5.3. Esketamine in TRD with Comorbid PTSD or Trauma-Memory Symptoms

There is limited but clinically informative evidence for intranasal esketamine in patients with treatment-resistant depression (TRD) and comorbid PTSD. Rothärmel et al. reported an open-label single-arm pilot study of 11 patients with TRD and comorbid chronic PTSD, with improvement in depressive symptoms and no serious adverse reactions (Rothärmel et al., 2022). A separate multicentre retrospective case series examined 22 patients with TRD and comorbid PTSD who experienced at least one trauma re-experiencing episode during esketamine sessions across multiple psychiatric departments (Rothärmel et al., 2026). In most cases, these episodes resolved as the sessions progressed. When treatment continued, a clinical response was observed for both depression and PTSD.

These data are directly relevant to safety planning but should not be described as an extended follow-up of the pilot study unless identical cohort linkage can be documented. Additional feasibility data from repeated oral esketamine in TRD with comorbid PTSD suggest improved resilience, affect regulation, and receptiveness to psychotherapy (Veraart et al., 2023). Two phenomenological case studies in depersonalized depression also support the hypothesis that esketamine-induced disembodiment may create a temporary window of psychological accessibility (Sarasso et al., 2024). Nonetheless, these heterogeneous feasibility signals provide a preliminary empirical rationale for testing the proposed protocol under controlled conditions. They do not establish efficacy of intranasal esketamine for trauma-memory symptoms, nor do they demonstrate that esketamine specifically augments psychotherapy.

### 5.4. Intranasal Esketamine or Nasal Ketamine Combined with Psychotherapy in PTSD

Currently, evidence directly combining intranasal esketamine or nasal ketamine with structured psychotherapy for PTSD remains limited to small uncontrolled studies. Roullet et al. retrospectively described six patients receiving intranasal esketamine alongside heterogeneous psychotherapies in a clinical setting, with improvement in depressive and PTSD symptoms but substantial interpretive limits due to the uncontrolled design and intervention heterogeneity (Roullet et al., 2025). Rohde et al. reported a pilot case series of three patients with chronic, treatment-resistant PTSD receiving nasal ketamine plus trauma-focused psychotherapy over eight weeks, with clinically relevant symptom reductions and good short-term tolerability (Rohde et al., 2024). These studies support feasibility, but not efficacy, and underscore the need for standardised psychotherapy content and prospective controlled designs. At present, they cannot determine whether observed improvements reflect the pharmacological intervention, the psychotherapy, nonspecific clinical support, or their interaction. Key studies are summarized in Table 1.

## 6. Feasibility-Oriented Protocol Proposal: Integrating Intranasal Esketamine and Traumatic-Memory Psychotherapy in TRD

### 6.1. Rationale and Objectives

The reviewed literature suggests that ketamine-class compounds can reduce PTSD symptoms in some settings and that combination approaches with psychotherapy are feasible in small preliminary studies. However, evidence is sparse for intranasal esketamine specifically, and even more limited for standardised protocols targeting trauma memories in TRD populations. TRD patients with clinically relevant trauma-memory symptoms are therefore best viewed as a priority population for exploratory research, not as a population in which efficacy can presently be assumed. The potential rationale is that rapid antidepressant effects could reduce avoidance behaviors and increase the ability to engage in trauma-focused interventions.

The primary objective is to evaluate the feasibility, acceptability, and safety of integrating intranasal esketamine with a structured traumatic-memory psychotherapy module in TRD patients with clinically relevant trauma-memory symptoms. The secondary objective is to estimate preliminary signals regarding depressive symptoms, frequency and distress of trauma-memory intrusions, and PTSD symptom clusters, with the explicit understanding that the study is not designed to provide confirmatory evidence of efficacy.

### 6.2. Study Design and Setting

We propose a two-phase research plan. Phase 1 is a single-arm feasibility pilot (*N* = 12; initial recruitment target *N* = 15 with a 20% attrition buffer) designed to refine procedures, assess adverse-event incidence, and collect preliminary acceptability data for two alternative psychotherapy-timing models (see Section 6.4). This is consistent with standard pilot-study methodology, which prioritises procedural refinement and feasibility assessment before definitive testing (Lancaster et al., 2004; Thabane et al., 2010). Participants will be assigned sequentially: the first approximately 6 participants (with buffer 7–8) to Model A (psychotherapy 24–48 h post-dose) and the next approximately 6 participants (with buffer 7–8) to Model B (psychotherapy within 120 min post-dose). This sequential allocation is pragmatic and intended for descriptive feasibility assessment only, not for inferential comparison between timing models. At the end of Phase 1, the safety, tolerability, attendance and acceptability data will be reviewed descriptively according to the timing model, in order to select a single model for Phase 2. A timing model will be deemed unsuitable for continuation if it is associated with any of the following:

(i) a timing-related serious adverse event;

(ii) recurrent, clinically significant flashbacks, or delayed destabilisation requiring unscheduled clinical intervention in two or more participants;

(iii) conversion of more than 25% of planned trauma-processing sessions to stabilisation/supportive-only sessions, because readiness criteria were not met;

(iv) psychotherapy attendance below 80%, attributable to timing;

(v) consistently poor acceptability ratings from participants or therapists. These are descriptive feasibility decision rules, not inferential comparison criteria. If neither model is clearly favoured, Model A will be adopted as the default because it avoids trauma processing during peak pharmacological dissociation.

Phase 2 is a randomised controlled pilot trial comparing two parallel groups: Arm 1 (control), standard intranasal esketamine care according to approved clinical protocols; Arm 2 (experimental), the same esketamine protocol plus structured imagery rescripting using the timing model selected in Phase 1. A placebo arm was not included at this stage because esketamine is an approved treatment for TRD, psychotherapy allocation cannot be blinded, and the purpose of Phase 2 is feasibility and preliminary effect-size estimation rather than confirmatory efficacy testing. Accordingly, this two-phase study is not statistically powered to test efficacy. Consistent with this exploratory aim, a formal confirmatory sample-size calculation for Phase 2 is not claimed at this stage; feasibility and variance estimates from Phase 1 would inform the design of any subsequent definitive trial. Because psychotherapy cannot be blinded and no attention-matched psychotherapy control is included, any between-arm differences in Phase 2 would be interpreted as preliminary and potentially confounded by nonspecific therapeutic contact, expectancy, and therapist effects. Randomization in Phase 2 will use a computer-generated allocation sequence with concealed allocation administered by an independent data manager. A future definitive trial could incorporate an active control condition, such as esketamine plus supportive counseling of matched duration. The protocol is designed to be implemented in a certified esketamine clinic with established monitoring procedures, access to clinicians trained in trauma-focused psychotherapy, and an integrated, multidisciplinary care structure incorporating psychiatric, psychotherapeutic, and nursing roles (Martiadis et al., 2026).

### 6.3. Participants

The suggested inclusion criteria are: adults (aged 18–70 years) who meet the criteria for TRD (non-response to ≥2 adequate antidepressant trials) and who are eligible for intranasal esketamine according to local regulatory and clinical standards (Food and Drug Administration, 2025). These adults must present with clinically significant trauma-related symptoms, which can be categorised as either: (a) current DSM-5 PTSD, confirmed clinically and, where feasible, supported by CAPS-5; or (b) subthreshold PTSD/trauma-related symptoms, defined as recurrent memories or flashbacks of an identifiable traumatic event, associated avoidance and clinically significant distress or impairment in functioning. Eligibility under criterion (b) should be supported by pre-specified self-report trauma thresholds (e.g., PCL-5 or IES-R), a brief intrusion diary and exact numerical cut-offs specified in the study manual.

Suggested exclusion criteria include: unstable medical conditions that contraindicate esketamine; uncontrolled hypertension (systolic > 180 mmHg or diastolic > 110 mmHg); current psychotic disorder or active manic episode; active substance use disorder (other than nicotine) within the past three months; acute suicide risk requiring a higher level of care; active dissociative disorder of a severity that precludes informed consent; inability to engage in collaborative safety planning.

### 6.4. Intervention Components and Timing

The esketamine component involves administering intranasal esketamine at standard clinical doses, in line with the locally approved prescribing information.

All participants will start with 56 mg per session and, at the supervising clinician’s discretion, the dose can be increased to 84 mg following standard titration criteria (tolerability and clinical response) and in line with the approved prescribing information. Administration will follow standard protocols, including mandatory post-dose observation periods and comprehensive safety monitoring (Food and Drug Administration, 2025). This includes an induction phase of twice-weekly dosing for four weeks, followed by maintenance as clinically indicated. The psychotherapy component is a structured, trauma-focused module designed to update intrusive memory representations and their associated meanings. Imagery Rescripting (ImRs) has been designated the sole standardised psychotherapy module for this protocol because it directly targets intrusive memory representations and their associated meanings. It offers a manualised procedure for updating memories and can be delivered in a relatively titratable format (Kip et al., 2023). ImRs was primarily selected for standardisation and evaluability in an early feasibility study; it has not been established that it is superior to prolonged exposure, written exposure therapy, EMDR, cognitive processing therapy or TIMBER-like approaches in this setting. Compared with more exposure-intensive approaches, ImRs may allow for more flexible titration in patients with severe depression, dissociation, or complex comorbidity. To preserve intervention standardisation, these alternative trauma-focused modalities were not incorporated into the present protocol, although they may be examined in future protocol variants. Therefore, its fit with the proposed esketamine-related window is conceptual and hypothesis-based rather than evidentiary.

All therapists delivering the ImRs module must have completed a recognised ImRs training programme and supervised a minimum of five ImRs cases prior to participating in the study. Fidelity monitoring will consist of: (i) audio recording of all psychotherapy sessions with participant consent; (ii) independent fidelity rating of a randomly selected ≥25% sample of sessions using a validated ImRs adherence checklist; (iii) monthly group supervision for all study therapists throughout the trial. Audio recordings will be pseudonymised and stored in encrypted form on access-restricted institutional servers. They will be used solely for fidelity monitoring and retained or destroyed according to institutional data protection policy.

**Figure 1:**
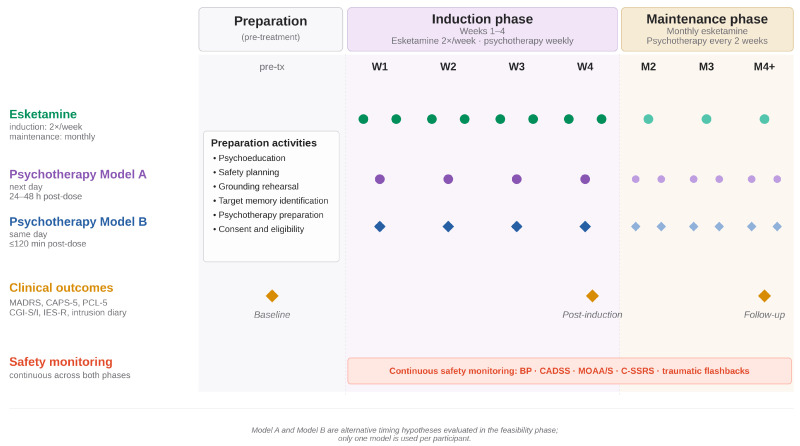
Proposed protocol schema for integrating intranasal esketamine and traumatic-memory psychotherapy in TRD. The preparation phase (column 1) includes all pre-treatment activities and the baseline assessment. During the induction phase (weeks 1–4), intranasal esketamine is administered twice weekly, and one structured psychotherapy session per week is scheduled using one of two timing models: Model A (24–48 h post-dose, next day) or Model B (same-day, <=120 min post-dose). Assessment timepoints (amber diamonds) occur at baseline, post-induction, and follow-up and include clinical outcome measures (MADRS, CAPS-5, PCL-5, CGI-S/CGI-I, IES-R) and trauma-memory intrusion monitoring. Safety monitoring (BP, CADSS, MOAA/S, C-SSRS, traumatic flashbacks) occurs continuously throughout both phases. The maintenance phase involves monthly esketamine and biweekly psychotherapy. In Phase 1, Models A and B are evaluated sequentially for descriptive feasibility only; a single model is then carried forward into Phase 2. BP = blood pressure; CADSS = Clinician-Administered Dissociative States Scale; CAPS-5 = Clinician-Administered PTSD Scale for DSM-5; CGI-S/CGI-I = Clinical Global Impression-Severity/Improvement; C-SSRS = Columbia Suicide Severity Rating Scale; IES-R = Impact of Event Scale-Revised; MADRS = Montgomery-Åsberg Depression Rating Scale; MOAA/S = Modified Observer’s Assessment of Alertness/Sedation; PCL-5 = PTSD Checklist for DSM-5; TRD = treatment-resistant depression.

The timing hypothesis is that psychotherapy sessions should be scheduled in close temporal proximity to esketamine administration. Two timing models may be evaluated during the feasibility phase: (a) Next-day trauma-memory psychotherapy (Model A), which occurs within 24–48 h of dosing when the effects of acute sedation and dissociation have resolved, but the effects of neuroplasticity may still be active; and (b) Late-session psychotherapy (Model B), which occurs approximately 90–120 min post-dose and focuses on memory updating once the patient is clinically stable. The proposed protocol schema is illustrated in Figure 1.

Preparation and integration: prior to the first esketamine session, all participants receive structured psychoeducation, grounding technique rehearsal, and collaborative identification of a target trauma memory. Each psychotherapy session includes: (i) a grounding and safety check; (ii) a structured memory updating or rescripting, and (iii) an integration and relapse-prevention planning. A brief post-session check-in should be conducted within 24 h of any session in which trauma material was actively processed.

### 6.5. Outcomes and Measures

Feasibility outcomes include recruitment and retention rates at the study endpoint, attendance rates for esketamine and psychotherapy sessions, participant acceptability ratings, and therapist-rated fidelity to the psychotherapy protocol.

Safety outcomes include esketamine-related adverse events (e.g., sedation as measured by the Modified Observer’s Assessment of Alertness/Sedation [MOAA/S] (Chernik et al., 1990), dissociation severity as assessed by the Clinician-Administered Dissociative States Scale [CADSS] (Bremner et al., 1998), and blood pressure changes); the occurrence and severity of distressing traumatic flashbacks during or within 48 h of dosing; and the emergence or worsening of suicidal ideation or self-harm behavior as assessed by the Columbia Suicide Severity Rating Scale (C-SSRS) (Posner et al., 2011). These outcomes are stratified by relevant clinical moderators, including gender (Martiadis et al., 2025).

Exploratory clinical outcomes include depression severity (Montgomery-Åsberg Depression Rating Scale [MADRS] (Montgomery & Åsberg, 1979) as the primary depression endpoint, with the Quick Inventory of Depressive Symptomatology [QIDS] (Rush et al., 2003) as a secondary self-report measure), PTSD severity (Clinician-Administered PTSD Scale for DSM-5 [CAPS-5] (Weathers et al., 2013a) as the primary PTSD endpoint, with the PTSD Checklist for DSM-5 [PCL-5] (Weathers et al., 2013b) administered at each session for repeated measurement), trauma-memory intrusion frequency and distress as assessed by a validated daily intrusion diary, and global clinical severity (Clinical Global Impression-Severity and Improvement scales [CGI-S/CGI-I] (Guy, 1976). Anhedonia should be assessed as a secondary outcome using the MADRS anhedonia subscale items. The Impact of Event Scale-Revised (IES-R) (Weiss & Marmar, 1997) may be used as an additional self-report measure of trauma-related distress. Where feasible, ecological momentary assessment (EMA) via smartphone could provide ecologically valid detection of day-to-day changes in intrusion burden.

### 6.6. Data Analysis

For the feasibility pilot, analyses should emphasize descriptive metrics, including recruitment rate (participants enrolled per month), retention rate (proportion completing the full protocol), session attendance (proportion of scheduled esketamine and psychotherapy sessions attended), and adverse event incidence, each reported with exact 95% confidence intervals. Pre-specified feasibility success criteria should be defined a priori (e.g., >=70% retention, >=80% session attendance). For Phase 2, the emphasis should remain on estimation rather than formal hypothesis testing. Linear mixed-effects models with random intercepts and slopes may be used to describe within-group and between-group symptom trajectories over time (baseline, mid-induction, post-induction, and follow-up) while accommodating missing data under a missing-at-random assumption, consistent with intent-to-treat principles. The primary depression outcome (MADRS) and primary PTSD outcome (CAPS-5) should be analyzed separately. Effect sizes (Cohen’s d) with 95% confidence intervals should be reported for all clinical outcomes to inform sample size calculations for a future definitive trial. This is consistent with recommendations for pilot randomized trials, in which variance and effect-size estimates are used to inform the design of a later definitive study (Whitehead et al., 2016). Because only one timing model will be carried forward into Phase 2, no inferential comparison between Model A and Model B is planned. In Phase 1, timing-model data will be summarized descriptively only. The following will be reported by model: session attendance, participant acceptability ratings, therapist-rated feasibility, adverse-event incidence, and flashback frequency/severity. These descriptive summaries will inform the selection of the Phase 2 timing model when interpreted according to the pre-specified feasibility decision rules described in Section 6.2. They should not be interpreted as comparative efficacy evidence.

Sensitivity analyses may include per-protocol analyses and assessment of the impact of baseline trauma severity on treatment response.

## 7. Ethical and Safety Considerations

Combined esketamine–psychotherapy protocols require a comprehensive, multi-component safety framework that addresses the distinct risk profiles of each intervention and their potential interactions.

### 7.1. Regulatory and Ethical Oversight

All research under this protocol requires prospective review and approval by an accredited Institutional Review Board (IRB) or Ethics Committee. Written informed consent, including discussion of the specific risks associated with esketamine (e.g., sedation, dissociation, blood pressure changes and potential for misuse), psychotherapy (e.g., transient distress and emotional flooding) and the combination of the two is mandatory for all participants. For Model B, consent for same-day trauma memory processing must be obtained during the preparation phase, prior to any dosing session. On dosing days, participants must reaffirm their willingness to proceed only after they meet the protocol’s predefined clinical stability criteria. No new consent for trauma processing will be sought during intoxication or residual dissociation.

Consent is an ongoing process and participants may withdraw at any time without any consequences for their clinical care.

### 7.2. Data Safety Monitoring Board

Prior to the commencement of Phase 1, an independent Data Safety Monitoring Board (DSMB) will be constituted. The DSMB will comprise a senior psychiatrist, a clinical psychologist experienced in trauma-focused research and a biostatistician, none of whom are affiliated with the study team. The DSMB will review all safety data quarterly and at pre-specified interim points (after the first six participants in Phase 1 and after fifteen participants in Phase 2). The DSMB has the authority to recommend protocol modification or termination at any time.

### 7.3. Adverse Event Monitoring and Recording

All adverse events (AEs) and serious adverse events (SAEs) will be systematically recorded by the supervising psychiatrist at each session and at all follow-up contacts, and will be rated using the Common Terminology Criteria for Adverse Events (CTCAE v5.0). AEs will be categorised as related to esketamine administration or the psychotherapy session, and reported to the DSMB and, as required, the Ethics Committee.

### 7.4. Esketamine-Specific Safety Monitoring

During and after each esketamine session, a qualified nurse or physician will conduct standardised monitoring: (i) blood pressure and heart rate at pre-dose, 40 min, and 120 min post-dose; dosing will be suspended if systolic blood pressure exceeds 180 mmHg or diastolic blood pressure exceeds 110 mmHg; (ii) MOAA/S assessment at 40 and 90 min post-dose to quantify sedation; (iii) CADSS assessment at 40 min and again immediately before any same-day psychotherapy session; (iv) C-SSRS at each visit; and (v) standardised documentation of traumatic flashbacks during and within 48 h of dosing. For Model B sessions, memory-focused psychotherapy may begin only if the participant is clinically stable, defined operationally as MOAA/S = 5 and CADSS total score < 15 at the pre-psychotherapy assessment. If MOAA/S < 5 or CADSS ≥ 15, trauma-focused work will not proceed and the session will be converted to supportive monitoring and grounding only. The CADSS threshold used here should be understood as a pragmatic, conservative readiness rule for this feasibility-oriented protocol, rather than as a validated, psychotherapy-specific cut-off point.

Participants at elevated baseline risk (identified by prior adverse reaction to esketamine, history of severe dissociation, or active suicidal ideation at screening) will receive additional post-session telephone contact and mandatory senior psychiatric review prior to each session. These measures will supplement, but not replace, the standardised monitoring procedures applied to all participants.

### 7.5. Psychotherapy-Specific Safety Monitoring

Risks associated with psychotherapy include transient increases in distress during trauma-memory processing, acute emotional flooding, dissociative destabilisation, and, in rare cases, symptom decompensation. These risks will be managed through: (i) mandatory pre-session grounding and safety checks; (ii) pre-specified criteria for pausing or suspending trauma processing. Trauma-focused work will not start, or will be immediately suspended, if any of the following occur: (a) Subjective Units of Distress (SUD) > 8/10 sustained for more than 10 min despite standardised grounding; (b) CADSS total score ≥ 15 or a clinically significant escalation of dissociation during the session; (c) inability to establish or maintain a grounding anchor; (d) the participant asks to stop trauma processing at any time; or (e) MOAA/S < 5 or any other sign of insufficient clinical stability. In these situations, the session will be converted to stabilisation/supportive work only, and resumption of trauma processing will require reassessment at a subsequent visit; (iii) graded and collaborative engagement with trauma material, with explicit patient control over pace and depth of processing; (iv) structured post-session stabilisation and psychoeducation; and (v) mandatory check-in contact within 24 h of any session involving active trauma processing. Any delayed destabilisation, severe recurrence of flashbacks, or emergence or worsening of suicidal thoughts outside the session window will trigger a same-day senior psychiatric review and, when necessary, an urgent in-person assessment. These criteria are pre-specified in the study manual. Clinician judgment operates as an additional safety override, not as a substitute for the standardised stopping rules.

### 7.6. Stopping Rules

Pre-specified stopping rules include: (i) the occurrence of two or more SAEs that are possibly related to the combined intervention; (ii) a DSMB recommendation following any interim safety review; (iii) failure to meet the pre-specified feasibility threshold of ≥70% retention at the midpoint of Phase 1.

### 7.7. Location and Clinical Oversight

The protocol will be conducted exclusively within a certified esketamine clinic with on-site medical coverage throughout all esketamine administration and the mandatory post-dose observation period. A senior supervising psychiatrist must be available on site during all sessions. Psychotherapy sessions conducted outside the immediate post-dose window (Model A) must take place within the same clinical facility or an adjacent setting with direct access to psychiatric support. In line with standard esketamine precautions, all participants must have pre-arranged transport home and must not drive or travel alone until the following day. Those living alone or deemed to be at a higher risk should receive follow-up contact on the same evening, a clinical check-in the next day, and documented emergency escalation instructions.

## 8. Discussion

### 8.1. Synthesis and Critical Appraisal of the Evidence

This narrative review identified converging but still preliminary evidence suggesting that ketamine-class compounds may alleviate PTSD symptoms and may support engagement in trauma-focused psychotherapy under some conditions. Three randomised controlled trials of intravenous ketamine demonstrated rapid and clinically meaningful reductions in PTSD symptom severity in adults with chronic PTSD (Feder et al., 2014, 2021; Abdallah et al., 2022). These are the strongest controlled data in the field to date. However, the magnitude and durability of these effects must be contextualized: response rates were not uniformly high, follow-up periods rarely extended beyond two to four weeks, and none of the trials were designed to determine whether symptom change reflected durable modification of traumatic-memory mechanisms. Thus, even the intravenous ketamine literature supports proof-of-concept more than a settled mechanistic or clinical model.

Studies combining ketamine with structured psychotherapeutic approaches represent an important methodological evolution (Shiroma et al., 2020; Feder et al., 2025; Topel & Ciccone, 2025). However, the majority of these studies have open-label or uncontrolled designs and small sample sizes, which makes it difficult to isolate the specific contribution of ketamine. Therapeutic alliance, expectancy effects and the broader context of intensive clinical attention may also influence outcomes. A useful precedent can be found in the literature on MDMA-assisted psychotherapy, where Phase 3 randomised trials demonstrated the pharmacological augmentation of manualised therapy delivered in an identical therapeutic context (Mitchell et al., 2021). Analogous controlled designs are now required in the ketamine/esketamine field before causal claims can be made.

Specifically with regard to the use of intranasal esketamine, the evidence base for its combination with psychotherapy remains very limited. It consists of pilot data (Rothärmel et al., 2022), retrospective safety and clinical case series (Roullet et al., 2025; Rohde et al., 2024; Rothärmel et al., 2026), and qualitative reports on patient experience (Sarasso et al., 2024; Olivola et al., 2026). While these reports are useful for generating hypotheses and planning safety, they do not support efficacy claims or demonstrate that esketamine enhances psychotherapy. At most, they demonstrate that such combinations can be delivered within existing esketamine care infrastructures (Martiadis et al., 2026) and that patient experience in this setting is clinically heterogeneous.

### 8.2. The Plasticity Window Hypothesis: Mechanistic Plausibility and Open Questions

As outlined in Section 4, the proposed protocol is informed by the concept of a ‘plasticity window’, which should be understood as a working model rather than an established clinical mechanism. The key question for the present protocol is whether the effects of esketamine-related plasticity can be translated into a time-limited, clinically meaningful opportunity for updating trauma memories within psychotherapy. In memory science, reconsolidation is one relevant, albeit incomplete, mechanistic explanation for this phenomenon. Reactivated memories may transiently return to a labile state, and NMDA-dependent processes appear to contribute to their re-stabilisation (Nader et al., 2000). This offers a conceptual rationale for pairing esketamine administration with controlled trauma-memory retrieval. If a psychotherapy session activates a target traumatic memory during or shortly after esketamine administration, the pharmacological effects on NMDA-dependent processes could influence how that memory is stabilised, potentially modifying its emotional significance or meaning. In the present clinical context, however, this remains a theoretical hypothesis rather than a demonstrated mechanism.

However, several critical uncertainties must be acknowledged. First, the reconsolidation window in humans is less well characterized than in rodent models, and the conditions under which clinically meaningful reconsolidation interference can be achieved in complex, multidimensional traumatic memories versus simple conditioned fear stimuli remain debated. Second, the esketamine dose used for antidepressant purposes may not produce the same degree of NMDA blockade as the subanesthetic infusion doses studied in reconsolidation research. Third, the proposed timing models (Model A: 24–48 h post-dose; Model B: <=120 min post-dose) are based on theoretical extrapolations rather than established human pharmacokinetic-pharmacodynamic data for reconsolidation-relevant targets. These uncertainties are precisely why both timing models are presented as empirical hypotheses to be explored descriptively during the feasibility phase rather than as established clinical recommendations.

### 8.3. Positioning Within the Landscape of Medication-Enhanced Psychotherapy

The proposal to combine esketamine with trauma-focused psychotherapy falls under the broader umbrella of medication-enhanced psychotherapy. The most developed comparator in trauma treatment is MDMA-assisted psychotherapy. A Phase 3 randomised trial demonstrated that, compared with therapy plus placebo, MDMA combined with manualized psychotherapy produced greater PTSD symptom reduction and functional recovery (Mitchell et al., 2021). This comparison is methodologically informative but not evidentiary for esketamine, given the distinct pharmacology, indications, regulatory contexts, and psychotherapeutic models involved.

Esketamine occupies a distinctive niche in this landscape. Unlike MDMA or psychedelics, esketamine is already approved and routinely administered in certified clinical settings for treatment-resistant depression (TRD), with established safety monitoring protocols, mandatory post-dose observation periods, and trained multidisciplinary teams (Martiadis et al., 2026). This existing infrastructure may lower the implementation barrier for adding a structured psychotherapy module compared with building an entirely new clinical environment from scratch. Furthermore, the TRD population, which is the current approved indication for esketamine, has a high prevalence of trauma exposure and PTSD comorbidity, meaning an esketamine clinic may already be treating individuals who could be candidates for a combined approach. This is a practical advantage, but not evidence that the model is clinically effective.

It is also worth noting that esketamine’s rapid antidepressant effects may overcome a particular obstacle to psychotherapy engagement that MDMA and psychedelics do not directly address severe anhedonia and psychomotor retardation. In treatment-resistant depression (TRD), these symptoms may specifically impair the motivational and engagement resources required for active participation in trauma-focused work. Therefore, the reduction in anhedonic symptoms with repeated esketamine administration (D’Andrea et al., 2025) could function not only as a direct symptom benefit, but also as a prerequisite for effective psychotherapeutic engagement—a pharmacological scaffold that enables psychological work to proceed.

### 8.4. Novelty and Potential Contribution of the Proposed Protocol

The protocol proposed in this paper is intended to contribute to the emerging and currently fragmented literature in several ways, while remaining exploratory in purpose. First, it specifies a standardised, manualized psychotherapy module centered on imagery rescripting. This directly addresses a major weakness of existing combined studies: the absence of a reproducible and evaluable psychotherapy component. Standardization is necessary if future studies are to examine whether psychotherapy adds value to esketamine alone and whether timing or patient characteristics moderate outcomes.

Second, the protocol operationalizes two competing timing hypotheses (Model A and Model B) within a single feasibility design. This permits a structured descriptive evaluation of their safety and acceptability profiles before one timing model is fixed for a later pilot RCT; it is not intended as a powered comparative test. This approach is methodologically efficient and directly addresses the current evidence gap, in which multiple timing strategies have been employed across studies without comparative evaluation.

Third, the protocol explicitly targets TRD patients with clinically significant trauma-memory symptoms, who are currently underserved by pharmacological and psychotherapeutic treatments. Standard TRD pharmacological protocols do not address trauma-memory mechanisms, and standard trauma-focused psychotherapy trials often exclude patients with severe depressive episodes. The proposed combined model is therefore clinically relevant, although it remains untested.

Fourth, the protocol is designed for implementation in certified esketamine clinics with established, integrated, multidisciplinary care frameworks. This avoids the need to create entirely new clinical environments. The pragmatic design may increase feasibility and eventual real-world applicability, although generalizability cannot be assumed until the model has been tested prospectively.

Fifth, the protocol incorporates systematic assessment of trauma-memory-specific outcomes, including intrusion frequency, distress during memory retrieval, and avoidance, alongside standard depression and PTSD measures. This outcome specification may help explore whether observed changes are more consistent with trauma-memory updating or with broader antidepressant effects alone. Distinguishing between these possibilities is essential for future mechanism-focused trials.

### 8.5. Clinical Messages

For clinicians operating esketamine programs, encountering patients with TRD and significant trauma histories is common, given the high co-prevalence of trauma and treatment resistance (Fantasia et al., 2025). This synthesis suggests several practical considerations. Trauma-memory symptoms should be assessed systematically before and during esketamine treatment using validated instruments such as the PCL-5 or the Impact of Event Scale-Revised. Intrusions, flashbacks, or avoidance behavior emerging during or shortly after dosing should be treated primarily as clinically relevant events requiring monitoring, grounding, and supportive management. In some cases they may reflect activation of trauma-related material, but current evidence does not justify interpreting them as markers of therapeutic progress (Rothärmel et al., 2026). Proactive psychoeducation before the first dosing session is therefore essential. Qualitative evidence further suggests that clear preparation regarding the phenomenology of esketamine-induced states may reduce distress and improve patients’ ability to contextualize these experiences (Olivola et al., 2026).

The composition of the multidisciplinary team surrounding the esketamine program is clinically relevant. Clinics that already include psychologists or psychotherapists trained in trauma-focused modalities—as increasingly recommended in integrated esketamine care frameworks—may be better positioned to pilot a combined model. Where such expertise is not yet available, the preparation phase of the proposed protocol offers a practical framework for developing competencies and team training.

Attention to individual differences in risk profiles is equally important. Available data suggest that suicidal and self-harm responses to esketamine may differ by gender (Martiadis et al., 2025), and trauma histories are differentially distributed across sex and gender groups. All participants should undergo standardised safety monitoring at every session. Additional monitoring measures may appropriately supplement, but should not replace, the standard protocol for participants at elevated clinical risk.

Particular vigilance is needed during the 48 h post-dose window, as the risk of trauma-related intrusions and affective dysregulation may be heightened by the combined intervention. Throughout the entire process, patient autonomy and shared decision-making are foundational. The decision to engage in trauma-memory-focused work during or after esketamine treatment should be collaborative, explicitly informed, and always revisable. There should be pre-agreed and clearly communicated thresholds for pausing or modifying the psychotherapy component.

### 8.6. Limitations

This review has several limitations that should be considered when interpreting its findings. (i) The narrative design is appropriate for an emerging field with small, heterogeneous studies. However, it is subject to selection bias in identifying, including, and emphasizing articles. Without quantitative pooling, estimates of effect magnitude cannot be derived, and the synthesis may inadvertently overweight more recent or more accessible literature. (ii) The field is evolving rapidly, so relevant studies may have been published after the January 2026 search cutoff. An update to this synthesis will likely be needed soon. (iii) The literature on combined interventions remains small and heterogeneous, typically involving samples of fewer than 30 people, uncontrolled designs and substantial variation in population, dosage, timing and psychotherapy model. This precludes meaningful cross-study comparisons or meta-analytic pooling. (iv) The proposed protocol remains theoretical and has not been tested. All key feasibility parameters are currently uncertain. The timing hypotheses (Model A vs. Model B) are not based on human pharmacokinetic-pharmacodynamic data related to reconsolidation-relevant targets, which leaves a substantial translational gap between preclinical mechanistic science and the proposed clinical application. (v) The proposed design also has important internal-validity limitations: Phase 1 uses sequential rather than randomised allocation to timing models, psychotherapy in Phase 2 cannot be blinded, and no attention-matched psychotherapy control is included. These features are acceptable for a feasibility-oriented proposal, but they restrict causal inference and mean that any apparent between-group differences would remain preliminary. (vi) Although systematic review evidence supports imagery rescripting across multiple disorders, it has not been directly evaluated in combination with ketamine or esketamine. Whether its memory-updating mechanisms are specifically potentiated by esketamine’s NMDA antagonism or are instead facilitated by the broader antidepressant and anxiolytic treatment context remains an open empirical question.

### 8.7. Future Research Directions

The research agenda resulting from this review is ambitious but should proceed in staged fashion. In the near term, the priority should be a feasibility study capable of generating reliable estimates of recruitment, retention, safety events, acceptability of timing models, and preliminary signal sizes on depressive and trauma-memory outcomes. These data are necessary to design later controlled studies and to determine whether the proposed multi-component design is workable in practice. If major feasibility problems emerge, subsequent iterations may need to isolate one design variable at a time, for example, timing model, psychotherapy augmentation, or dosing strategy.

Subsequently, adequately powered randomised trials comparing esketamine plus manualized psychotherapy with standard esketamine care—and ideally with attention-matched psychotherapy controls—will be needed. These trials should include standardised psychotherapy procedures and active monitoring of trauma-memory-specific outcomes. In parallel, the field should explore whether different psychotherapy modalities (e.g., imagery rescripting, WET, EMDR, and CPT) show differential compatibility with esketamine’s neurobiological and phenomenological effects. This would have important implications for personalized treatment matching.

From a neurobiological perspective, integrating functional and structural neuroimaging at various time points before, during, and after the combined intervention, with sufficient resolution to detect changes in prefrontal-amygdala connectivity, hippocampal volume, and default mode network organization, would provide evidence relevant to the plasticity window hypothesis. Coupling these neuroimaging measures with EEG-based indices of synaptic plasticity and plasma BDNF levels would allow us to identify biomarkers that can predict and explain individual variation in treatment response.

At the clinical translational level, examining generalizability across different trauma types (e.g., childhood vs. adult-onset, single-incident vs. complex/repeated), cultural contexts, and healthcare systems is essential. Most existing studies are conducted in high-income Western settings with predominantly non-complex PTSD presentations. To capture the subjective meaning patients attribute to the combined experience, including the phenomenological dimension of esketamine-induced dissociative states, patient-reported outcome measures and qualitative methods should be systematically embedded in all future trials. Finally, health economic evaluations embedded within controlled trials will be necessary to inform commissioning decisions and position the combined model within the broader landscape of available PTSD and TRD treatments.

## 9. Conclusions

Trauma-related intrusive memories are a clinically relevant target across PTSD and depressive disorders, including TRD. Intranasal esketamine is an approved treatment for TRD and represents a plausible candidate context for testing whether structured traumatic-memory psychotherapy can be delivered safely and feasibly. However, current evidence does not establish that intranasal esketamine augments psychotherapy, nor does it confirm that mechanisms such as reconsolidation interference or a clinically usable ‘plasticity window’ are operative in this setting. The literature reviewed here supports mechanistic plausibility and preliminary feasibility more than clinical efficacy. The protocol proposed in this paper should therefore be read as a feasibility-oriented, hypothesis-generating framework for future controlled studies rather than as an efficacy model ready for clinical adoption. If subsequent trials confirm benefit, such an approach could become relevant for the large population living with TRD and trauma-related memories.

## Figures and Tables

**Table 1:** Key clinical studies relevant to ketamine/esketamine, PTSD, traumatic memories, and psychotherapy integration (non-exhaustive; ordered by route of administration and publication year).

First Author (Year)	Study Design & Population	Ketamine/Esketamine Intervention	Psychotherapy Framework	Key Findings Relevant to Trauma Memories/PTSD
Feder et al. (2014)	RCT; chronic PTSD	Single IV ketamine vs. active control (midazolam)	—	Rapid PTSD symptom reduction; first rigorous RCT evidence.
Shiroma et al. (2020)	Proof-of-concept; veterans with PTSD	Subanesthetic IV ketamine + trauma sessions	Prolonged Exposure (PE)	Feasibility demonstrated; symptom changes require confirmation in larger trials
Feder et al. (2021)	RCT; chronic PTSD	Repeated IV ketamine vs. active control	—	Sustained PTSD symptom reductions across multiple infusions; durability uncertain
Abdallah et al. (2022)	Double-blind RCT; veterans/military with PTSD	Dose-ranging IV ketamine vs. placebo	—	Dose-related PTSD reductions; supports dosing strategy development
Rothärmel et al. (2022)	Open-label single-arm pilot study; TRD with chronic PTSD	Intranasal esketamine	—	Preliminary feasibility and improvement in depressive/PTSD symptoms; uncontrolled design
Duek et al. (2023)	Randomised mechanistic pilot study; PTSD	Ketamine vs. midazolam after trauma-memory retrieval	Four-day trauma-focused psychotherapy 24 h later	Neural and clinical findings consistent with enhanced post-retrieval extinction
Veraart et al. (2023)	Case series; TRD with comorbid PTSD	Repeated oral esketamine	Psychotherapeutic framework	Improved resilience and receptiveness to psychotherapy; supports comorbidity-relevant feasibility
Rohde et al. (2024)	Pilot case series; chronic treatment-resistant PTSD	Nasal ketamine	Trauma-focused psychotherapy	Feasible combined approach with clinical improvements; limited by uncontrolled design
Feder et al. (2025)	Open-label pilot; chronic PTSD	IV ketamine in temporal proximity to therapy	Written Exposure Therapy (WET)	Feasible combined approach; preliminary PTSD symptom reductions; uncontrolled design
Roullet et al. (2025)	Retrospective case series	Intranasal esketamine in clinical setting	Heterogeneous psychotherapy approaches	Hypothesis-generating feasibility data; causal attribution limited by design and intervention heterogeneity
Topel and Ciccone (2025)	Retrospective clinical chart review; PTSD	Sublingual ketamine in repeated sessions	EMDR (ketamine-assisted EMDR)	Feasibility and symptom outcomes reported; highlights timing/setting considerations; requires replication
Rothärmel et al. (2026)	Retrospective multicentre case series; TRD with comorbid PTSD	Intranasal esketamine	—	Trauma re-experiencing episodes often resolved over sessions; informative for safety planning and feasibility, but uncontrolled and non-comparative

The strongest evidence across the studies summarised in this table derives from three intravenous ketamine randomised controlled trials (RCTs), whereas intranasal esketamine and psychotherapy combination studies remain predominantly pilot, retrospective or case-based, and should therefore be interpreted mainly as evidence to generate hypotheses and inform feasibility studies. RCT = randomised controlled trial; TRD = treatment-resistant depression; PTSD = post-traumatic stress disorder; IV = intravenous; WET = written exposure therapy; EMDR = eye movement desensitization and reprocessing.

## Data Availability

No new data were generated or analyzed in this study. All data supporting reported findings are contained within the cited published references.

## Abbreviations

The following abbreviations are used in this manuscript:

BDNF	Brain-derived neurotrophic factor
BP	Blood pressure
CADSS	Clinician-Administered Dissociative States Scale
CAPS-5	Clinician-Administered PTSD Scale for DSM-5
CGI-S/CGI-I	Clinical Global Impression–Severity/Improvement
C-SSRS	Columbia Suicide Severity Rating Scale
DSM-5	Diagnostic and Statistical Manual of Mental Disorders, 5th Edition
EMA	Ecological Momentary Assessment
EMDR	Eye Movement Desensitization and Reprocessing
IES-R	Impact of Event Scale–Revised
MADRS	Montgomery–Åsberg Depression Rating Scale
MOAA/S	Modified Observer’s Assessment of Alertness/Sedation
mTORC1	Mammalian target of rapamycin complex 1
NMDA	N-methyl-D-aspartate
PCL-5	PTSD Checklist for DSM-5
PE	Prolonged Exposure
PTSD	Post-traumatic stress disorder
QIDS	Quick Inventory of Depressive Symptomatology
RCT	Randomised controlled trial
TIMBER	Trauma Interventions using Mindfulness-Based Extinction and Reconsolidation
TRD	Treatment-resistant depression
TrkB	Tropomyosin receptor kinase B
WET	Written Exposure Therapy
